# Hypophosphatemia in Diabetic Ketoacidosis During Intensive Care Admission

**DOI:** 10.3390/medsci14020249

**Published:** 2026-05-12

**Authors:** Nicolas A. Sieben, Sebastiaan Blank, Alexis Tabah, Kyle C. White, Kevin B. Laupland, Felicity Edwards, Antony Attokaran, Stephen Luke, Aashish Kumar, Stephen Whebell, Dinesh Parmar, James McCullough, Peter Garrett, Mahesh Ramanan

**Affiliations:** 1 Mater Public Hospital, Brisbane, QLD 4101, Australia; 2 Royal Brisbane and Women’s Hospital, Metro North Hospital and Health Services, Brisbane, QLD 4029, Australia; 3 Intensive Care Unit, Cairns Hospital, Cairns, QLD 4870, Australia; 4 Intensive Care Unit, Redcliffe Hospital, Redcliffe, QLD 4020, Australia; 5 Faculty of Medicine, Mayne Academy of Critical Care, University of Queensland, St Lucia, Brisbane, QLD 4072, Australia; 6 Intensive Care Unit, Princess Alexandra Hospital, Woolloongabba, QLD 4102, Australia; 7 Faculty of Health, Queensland University of Technology, Brisbane, QLD 4000, Australia; 8 Intensive Care Unit, Rockhampton Hospital, The Range, QLD 4700, Australia; 9 Intensive Care Services, Mackay Base Hospital, Mackay, QLD 4740, Australia; 10 Intensive Care Unit, Logan Hospital, Logan, QLD 4131, Australia; 11 Intensive Care Unit, Townsville Hospital, Townsville, QLD 4814, Australia; 12 Adult Intensive Care Services, The Prince Charles Hospital, Chermside, Brisbane, QLD 4032, Australia; 13 Intensive Care Unit, Gold Coast University Hospital, Southport, Gold Coast, QLD 4215, Australia; 14 Intensive Care Unit, Sunshine Coast University Hospital, Birtinya, QLD 4575, Australia; 15 Intensive Care Unit, Caboolture Hospital, Metro North Hospital and Health Services, Brisbane, QLD 4510, Australia; 16 The George Institute for Global Health, University of New South Wales, Sydney, NSW 2000, Australia

**Keywords:** diabetes, diabetic ketoacidosis, electrolytes, intensive care, endocrine

## Abstract

**Introduction:** Diabetic ketoacidosis (DKA) is a common complication of diabetes mellitus characterized by metabolic acidosis, ketogenesis, hypovolemia, hyperglycemia, and electrolyte depletion. During treatment of DKA with intravenous fluids and insulin, some electrolyte disturbances can worsen. Hypophosphatemia is one such electrolyte disturbance that has not been well characterized in patients with severe DKA requiring Intensive Care Unit (ICU) admissions. This study sought to evaluate the incidence, severity, associations, and outcomes of hypophosphatemia in DKA. **Methods:** This retrospective multicenter study was conducted across DKA admissions to Queensland ICUs from 2016 to 2021. Adult patients (>18 years) requiring ICU admission for management of DKA were included in this study. Patients with DKA were stratified by lowest recorded phosphate level as: normal ≥ 0.80 mmol/L, mild 0.50–0.79 mmol/L, moderate 0.30–0.49 mmol/L and severe < 0.3 mmol/L. Patient demographics, comorbidities, ICU-related supports, and medications (including fluid, insulin administration, phosphate, and other electrolyte replacement) were collected. Univariate analysis was performed between hypophosphatemia severity and normophosphatemia subgroups to determine risk factors, outcomes, replacement, and progression of hypophosphatemia in the ICU. Phosphate replacement and administered insulin was compared to nadir serum phosphate level. Multivariate analysis and linear regression were performed to identify risk factors for the development of hypophosphatemia. **Results:** A total of 842 admissions of 669 unique patients due to DKA were included; 436 of 842 (51.8%) admissions maintained normophosphatemia in the ICU, while 220 (26.1%, n = 220/842) had mild hypophosphatemia, 124 (14.7%, n = 124/842) had moderate hypophosphatemia and 62 (7.4%, n = 62/842) had severe hypophosphatemia. Patients with higher BMI, higher APACHE II/III score, cerebrovascular disease and all blood gas parameters (excluding PaO_2_) were found to have more severe hypophosphatemia. Lower serum phosphate was associated with greater replacement and greater insulin administration per kilogram body weight. ICU length of stay, hospital length of stay and mortality were not affected by degree of hypophosphatemia (*p* > 0.05). Linear regression revealed that standard base excess was strongly associated with the development of hypophosphatemia (β = 0.02, 95% CI 0.01–0.02, *p* < 0.001). **Conclusions:** Increasing severity of hypophosphatemia was associated with increasing severity of DKA. Increased ICU length of stay was related to increased severity of hypophosphatemia.

## 1. Introduction

Diabetic ketoacidosis (DKA) is a common complication of type 1 diabetes mellitus (DM) and less frequently type 2 DM, characterized by ketone body production, hyperglycemia, hypovolemia and severe metabolic acidosis [1,2]. Severe metabolic acidosis during DKA is a common indication for Intensive Care Unit (ICU) admission for monitoring DKA resolution, but sometimes other invasive supports including dialysis, vasoactive support and mechanical ventilation may be required [3]. DKA patients requiring ICU face additional challenges with more severe ketoacidosis and hypovolemia compared to their non-ICU counterparts. Fluid resuscitation is one of the core components of DKA treatment to correct severe hypovolemia, while insulin infusions and electrolyte replacement are required to achieve ketone suppression, slow glucose control and maintain electrolyte levels [3,4,5]. Without careful titration of therapies, complications can occur, including cerebral oedema due to osmolality shifts, critically low phosphate or potassium levels and ongoing hypovolemia [6,7].

One common electrolyte disturbance is hypophosphatemia (defined as serum phosphate concentration ≤ 0.80 mmol/L) commonly seen within 24–48 h of ICU admission and occurring in up to 74% of DKA admissions [8]. The exact pathophysiology of hypophosphatemia in DKA is unknown but has been postulated to occur under similar mechanisms as refeeding syndrome and has been shown to correlate in severity to the degree of acidosis seen during DKA [8,9]. A sudden shift from ketotic metabolism to aerobic glucose-based metabolism after insulin exposure rapidly consumes phosphate [8,9]. Hypophosphatemia during DKA has not been well studied during ICU admissions, and current guidelines fail to mention routine phosphate replacement despite being recognized as a common complication [9]. Hypophosphatemia in DKA is similar to the general critically ill population, where it has been demonstrated that there is no universal agreement on when and how to treat hypophosphatemia in the ICU [10,11,12]. Although additive to previous studies, this study uniquely seeks to define hypophosphatemia mechanisms in DKA related to therapies provided.

Given that hypophosphatemia occurs commonly in DKA, that it is a readily correctable electrolyte disturbance, and that there is a lack of evidence-based guidelines to assist clinicians’ decision-making on phosphate replacement in DKA, we conducted this study to characterize the incidence, severity, associations, and outcomes of hypophosphatemia in DKA patients requiring ICU admission in Queensland, Australia [13].

## 2. Materials and Methods

Study Design

This is a large, multicenter, retrospective, data linkage study using routinely collected data from electronic healthcare records, benchmarking data submissions, and administrative data collections. Strengthening the reporting of observational studies in the epidemiology (STROBE) checklist was adhered to during this study (Supplemental Table S1).

Study Sites

The included study sites included 12 closed-model Intensive Care Units representing 5 tertiary ICUs, 3 outer metropolitan ICUs and 4 regional ICUs across Queensland, Australia. These centers encompass most of Queensland’s public hospitals and most of the state’s ICU capacity including neurosurgical, obstetric, cardiothoracic, trauma and general intensive care across metropolitan and regional centers. Included patients were admitted to these units from 1 January 2017 and 31 December 2021. Patients were included if their records were retrievable and if they were coded to have had a primary admission diagnosis of DKA (ICD 10-AM Codes: E10.10, E10.11, E11.10, E11.11 and E13.10) (Supplemental Figure S1). The potential for misclassification is noted and could not be interrogated given the nature of the dataset. Readmissions were treated as a unique admission if containing the relevant ICD code. Mortality outcomes were adjusted for readmissions using unique patients in lieu of admissions. Readmissions assume independence of the patient and represent a limitation of the dataset. Palliative patients and patients who proceeded to organ donation were excluded from analysis. Phosphate replacement was site-dependent.

Data Sources

Routine clinical data was obtained from eCritical Metavision (™) (iMDsoft, Boston, MA, USA) clinical information systems. Extracted data was validated before analysis of this study. Patient data was collected including: demographics (including age, weight, body mass index [BMI], patient comorbidities, admission to ICU source), illness severity (Acute Physiology and Chronic Health Evaluation [APACHE] II/III scores and APACHE diagnosis groups), general biochemistry or other bloods across ICU admission, ICU supports (including dialysis, mechanical ventilation, etc.), medications given (including intravenous [IV] fluid, per oral [PO] medication, vasoactive medication, insulin, electrolyte replacement, etc.), and treatment goals and outcomes (including ICU length of stay [LOS], mortality, hospital LOS, etc.).

Serum phosphate was analyzed to determine the presence of hypophosphatemia for each patient and the severity of hypophosphatemia. Patients were stratified from lowest recorded serum phosphate on each day as: normal > 0.8 mmol/L, mild 0.5–0.79 mmol/L, moderate 0.3–0.49 mmol/L, severe < 0.3 mmol/L and critical < 0.1 mmol/L [14]. Laboratory testing and reference ranges were standardized across all sites by the state-wide diagnostic pathology service.

Outcomes

The primary outcome of this study was the incidence of hypophosphatemia during ICU admission. Secondary outcomes included ICU and hospital LOS, hospital mortality, duration of hypophosphatemia and the amount of phosphate replacement per day of ICU admission.

Statistical Analysis

Descriptive statistics were expressed as frequencies and proportions for categorical variables and medians with interquartile ranges (IQRs) for continuous variables. All variables were screened for normality before statistical testing. Statistical significance (*p* < 0.05) was detected using non-parametric tests for continuous variables, including the Kruskal–Wallis test or Wilcoxon signed rank test, and for categorical variables Fisher’s exact test or Chi-squared analysis was used. For parametric variables, the T-test or Welch T-test was used for continuous data where appropriate between patient phosphate level groups. Parametric and non-parametric data were screened for before statistical testing. Missing data was not imputed, as complete analysis could not be computed with missing data. Demographics, phosphate replacement and outcomes of ICU admission were calculated for each hypophosphatemia subgroup. Daily replacement of phosphate was plotted against the daily lowest serum phosphate level with Locally Estimated Scatterplot Smoothing (LOESS) assuming a non-linear relationship of treatment provided and measured serum phosphate values. The average lowest daily phosphate level for each hypophosphatemia subgroup was plotted. A Kaplan–Meier curve was performed for the percent remaining in the ICU (including discharge or death) from time of admission for each hypophosphatemia subgroup. Linear regression (univariate and multivariate with a patient-level random effect to account for repeated admissions) was used to evaluate the association between acid base status on ICU admission, as expressed using Standard Base Excess (SBE), and early insulin dosage, with nadir serum phosphate concentration during ICU stay while controlling for important confounders. Co variates were chosen a priori based on the authors’ clinical judgement and included age, sex, weight, diabetes type and the Charlson Comorbidity Index (CCI) [4,8,15,16]. The standardized based excess was chosen as the acid–base parameter for inclusion in the regression modeling due to its independence from arterial partial pressure of carbon dioxide and expected strong collinearity with other acid–base variables such as pH and anion gap. Calibration was assessed through the visualization of calibration plots, which depicted observed versus expected nadir phosphate concentrations. Partial R^2^ (incremental) was calculated for each component during linear regression. Formal model diagnostics were performed, including residual versus fitted, normal Q-Q, random intercept Q-Q plot and scale-location to assess regression assumptions. Generalized variance inflation factors were used for assessment of multicollinearity. Heteroscedasticity was assessed using model diagnostic testing with inspection of residual plots. The Shapiro–Wilk test was used to assess normality and component plus residual plots for the assessment of linearity of continuous predictors.

Ethical Considerations and Software

Ethics was approved by Metro South Hospital and the Health Service Human Research Ethics Committee under ethics application HREC/2022/QMS/82024 with an individual waiver of consent. The datasets presented in this article are not readily available due to patient and cohort confidentiality. All calculation and data management were performed with R Version 4.5.0 (R Foundation for Statistical Programming, Vienna, Austria). R packages used for analysis included: tidyverse, lubridate, gtsummary, readxl, survival, data.table, ggpubr, gridExtra, sandwich, patchwork, lme4, lmertest, broom.mixed, performance, DHARMa and knitr.

## 3. Results

Demographics

There were 842 DKA admissions among 669 patients (173 re admissions) included in this study (Supplementary Figure S1); 436 of 842 (51.8%) admissions maintained normal serum phosphate in the ICU, while 220 (26.1%, n = 220/842) had mild hypophosphatemia, 124 (14.7%, n = 124/842) had moderate hypophosphatemia and 62 (7.4%, n = 62/842) had severe hypophosphatemia. Only four patients had a serum phosphate nadir of <0.1 mmol, of which one patient survived a ventricular tachycardia rhythm, but otherwise they were included in the severe (<0.3 mmol) hypophosphatemia subgroup. The median age of all the serum phosphate groups was 43 [IQR 27–56] years, and 54% (n = 455/842) of patients were female across severity groups (Table 1). Across all subgroups, 75.1% (n = 632/842) of patients had type 1 diabetes and 21.5% (n = 210/842) of patients had type 2 diabetes (Table 1). Lower arterial pH, more negative base excess, lower bicarbonate, increasing anion gap and increasing serum glucose levels were all significantly associated with the development of hypophosphatemia and previously known to be significant with worsening DKA (*p* < 0.001 for all) (Table 1).

Phosphate Replacement and Insulin Administration

**Figure 1:**
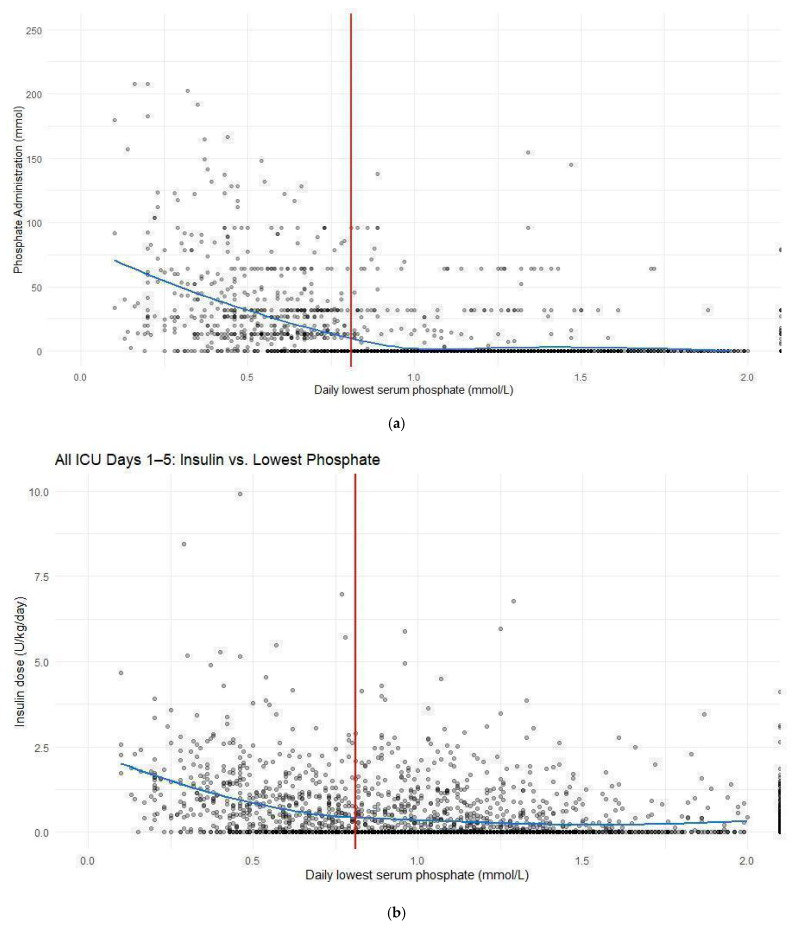
(**a**) The measured lowest daily phosphate level (mmol/L) versus the total daily phosphate replacement (mmol). The LOESS trendline is shown in blue. A vertical red line indicates a serum phosphate level of 0.81 mmol/L for the normal reference range. (**b**) The measured lowest daily phosphate level (mmol/L) versus the total daily insulin per unit weight administered (U/kg/day). The LOESS trendline is shown in blue. A vertical red line indicates a serum phosphate level of 0.81 mmol/L for the normal reference range.

The proportion of patients receiving phosphate replacement in hypophosphatemia subgroups was much higher for both IV (2.3% [normal serum phosphate] vs. 91.9% [severe] hypophosphatemia) and PO (1.8% [normal serum phosphate] vs. 54.8% [severe] hypophosphatemia) replacement (Table 2). Patients with an abnormal lowest serum phosphate level received both more total mean IV (0.4 ± 3.1 mmol normophosphatemia group vs. 74.0 ± 542.2 mmol for all patients with hypophosphatemia, *p* ≤ 0.001) across their respective admissions (Table 2). Patients with normal serum phosphate levels received less replacement than any hypophosphatemia groups on all days of admission (days 1–5) (Table 2). Daily replacement of phosphate was plotted against the serum phosphate level on that day in Figure 1a. Patients with lower serum phosphate levels received higher amounts of phosphate replacement (Figure 1a). Many patients continued to receive phosphate replacement despite normal or high serum levels (Figure 1a). This trend continued through days 1 through 5 of ICU admission (Supplemental Figure S2a–e). Some patients, despite having a normal phosphate level (>0.80 mmol/L) were observed to have phosphate replacement (Figure 1a). Insulin dosing was similarly compared to the daily minimum phosphate value, and patients receiving higher doses of insulin infusion had lower serum phosphate levels (Figure 1b). This trend continued through days 1 through 5 of ICU admission with reducing correlation as day number progressed (Supplemental Figure S2f–j).

Daily Serum Phosphate Levels

**Figure 2:**
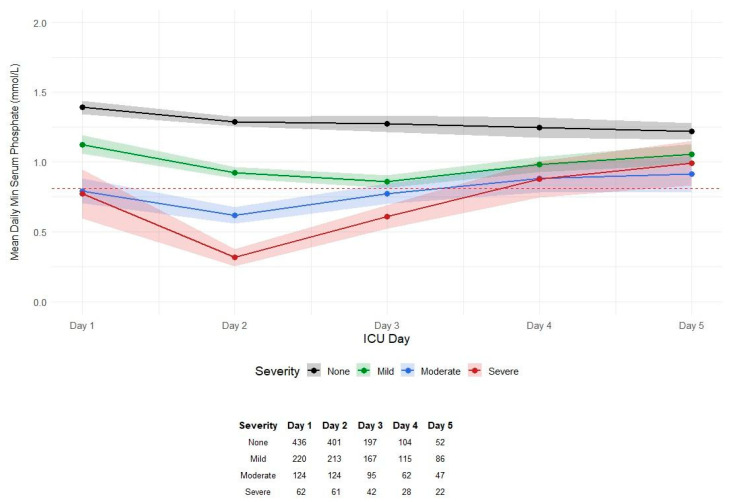
Daily average minimum serum phosphate levels (mmol/L) with 95% CI outlined by indicator color. A table below shows the total patient count in each subgroup for each day of ICU admission. A horizontal red line shows a serum phosphate level of 0.81 mmol/L for the normal reference range.

**Figure 3:**
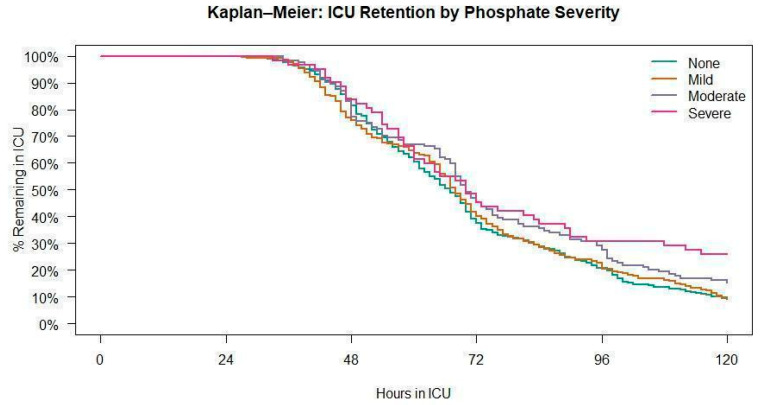
Kaplan–Meier curve with the remaining fraction of each hypophosphatemia or normophosphatemia subgroup remaining in the ICU from time of ICU admission.

All hypophosphatemia severity groups were able to achieve a normal mean serum phosphate level by day 4 of ICU admission, and only the mild hypophosphatemia group was able to achieve a normal mean serum phosphate by day 3 (Figure 2). Patients remaining in the ICU declined over the 5-day period, and patients with lower serum phosphate levels had longer ICU LOS (Figure 3). A Kaplan– Meier curve was performed and showed that ICU retention was dependent on the severity of hypophosphatemia (Figure 3).

Outcomes of hypophosphatemia

The outcome of each hypophosphatemia subgroup and the normophosphatemia group were collected (Table 3). All subgroups had a nadir of serum phosphate on day 1 (Table 3). The number of days and proportion of days with hypophosphatemia increased with the severity of DKA (Table 3). The ICU LOS and hospital LOS were not significantly different between subgroups (*p* > 0.05). Unadjusted outcomes such as ICU case fatality, hospital fatality, 30-day fatality and 90-day fatality were not significantly different between serum phosphate subgroups consistent with DKA fatality rates in general (Table 3).

**Figure 4:**
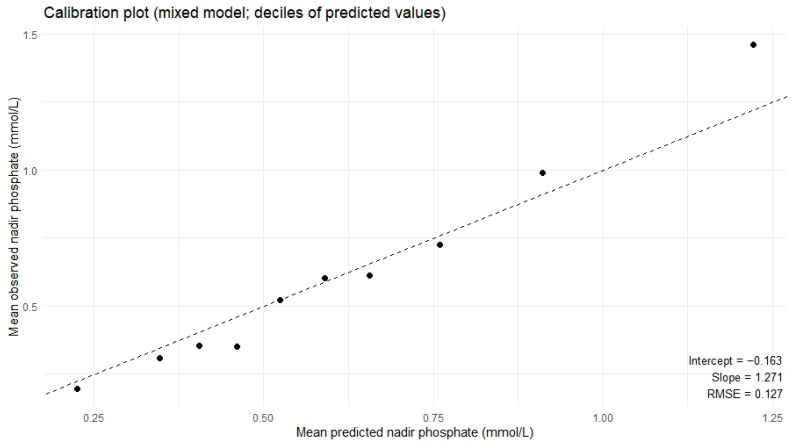
Calibration plot of the hypophosphatemia linear regression using the extended data set. Dashed line represents the line of perfect calibration. The black dots represent the predicted and observed values at each decile.

A linear regression was performed to investigate the association between baseline standard base excess and initial insulin dose with nadir serum phosphate concentration, adjusting for age, weight, diabetes type, and the Charlson Comorbidity Index. In univariate regression, both standard base excess (β = 0.02 per unit increase, 95% CI 0.01–0.03, *p* < 0.001) and insulin dose per hour (U/kg/h) (β = −0.13 per unit increase, 95% CI −0.24–−0.03, *p* = 0.01) were significant with respect to nadir serum phosphate. In multivariate regression (Table 4), baseline standard base excess was strongly associated with nadir phosphate concentration (β = 0.02 per unit increase, 95% CI 0.01–0.03, *p* < 0.001), whereas the initial insulin (U/kg/h) (β = 0.02 per unit increase, 95% CI −0.08–0.12, *p* = 0.72) dose was no longer significant for nadir serum phosphate in the multivariate analysis. Partial R^2^ analysis showed that standard base excess explained approximately 20% of the residual variability in nadir phosphate concentration, whereas other covariates each explained less than 5%. The calibration plot produced a linear trend with a −0.163 x-intercept, a slope equal to 1.27 and a root mean square error of 0.127, indicating a reasonably calibrated model (Figure 4). Model diagnostics were performed (Supplemental Figures S3 and S4, Supplemental Table S2). No major departures from regression assumptions were detected (Supplemental Figure S3). Heteroscedasticity was assessed and was not significant (*p* = 0.802, performance package R), and residual plots had minor tail deviation indicating no strong evidence of heteroscedasticity. The Shapiro–Wilk test suggested departure from residual normality (W = 0.905, *p* < 0.001). Taken together, these results suggest minor tail deviation rather than global model misspecification. Generalized variance inflation factors were low, indicating low multicollinearity (Supplemental Table S2). Component plus residual plots showed no marked non-linearity in continuous predictors (Supplemental Figure S4).

## 4. Discussion

### 4.1. Key Findings

This large, multicenter retrospective cohort study of 842 DKA admissions across 12 Queensland ICUs found that hypophosphatemia occurred in 48.2% of patients, with severe hypophosphatemia (<0.30 mmol/L) affecting 7.4% of admissions. No differences in hospital length of stay and mortality were observed. Standard base excess was strongly associated with hypophosphatemia development, presumably an epiphenomenon related to illness severity. Among the included variables, standard base excess accounted for the most variability in nadir phosphate concentrations (Partial R^2^ = 0.198). Phosphate nadir characteristically occurred on day 2 of ICU admission, with normalization achieved in all severity groups by day 4 with replacement therapy. Increasing hypophosphatemia severity was not significantly associated with longer ICU length of stay, hospital length of stay or mortality outcomes across severity subgroups.

### 4.2. Comparisons with Key Literature

The 48.2% incidence of hypophosphatemia in this cohort is lower than the 74% reported by van der Vaart and colleagues in their observational study of DKA patients, with both studies using the same threshold of 0.80 mmol/L for diagnosing hypophosphatemia [8]. This discrepancy may reflect differences in phosphate monitoring frequency, replacement practices, or population characteristics between studies. Notably, the current study included only ICU admissions representing more severe DKA presentations, whereas van der Vaart et al. included two general hospitals with a combined 132 admissions across 80 unique patients and generally had a younger population (mean 28.4 years). Our finding that the severity of metabolic acidosis (as reflected by standard base excess) correlates with hypophosphatemia development aligns with previous observations that acid– base disturbance severity is associated with electrolyte derangements in DKA [8,9].

The temporal pattern of hypophosphatemia observed in this study, with nadir phosphate concentrations occurring on day 2 of admission, differs from earlier hypotheses suggesting that phosphate depletion results primarily from rapid renal excretion to offset acidosis [8,9]. This delayed presentation provides stronger support for an insulin-mediated mechanism, wherein the metabolic shift from ketotic to glucose-based metabolism drives intracellular phosphate translocation for adenosine triphosphate production [8,17,18,19,20]. This pathophysiology closely parallels the refeeding syndrome, where insulin release following carbohydrate reintroduction precipitates similar intracellular electrolyte shifts [17,18,21,22].

The association between hypophosphatemia and prolonged ICU length of stay observed in our cohort is consistent with findings from the broader critical care literature. Attokaran et al. demonstrated similar associations in their multicenter study of hypophosphatemia across Queensland ICUs using the same ICU base population [10], while Wozniak and colleagues reported that hypophosphatemia on ICU admission was associated with increased duration of mechanical ventilation [23]. Sin et al. similarly identified phosphate abnormalities as predictors of adverse ICU outcomes in their retrospective multicenter analysis [11]. However, unlike studies in general critical illness populations, we did not observe mortality differences across phosphate severity groups, potentially reflecting the relatively young age, low mortality rates and low comorbidity burden typical of DKA populations, as well as the readily reversible nature of this electrolyte disturbance with appropriate replacement.

The lack of universal agreement on phosphate replacement thresholds and protocols in DKA, as highlighted by Ramanan et al. in their narrative review [12], is reflected in the variable replacement practices observed in this cohort. Current DKA guidelines fail to provide specific recommendations for routine phosphate monitoring and replacement despite recognizing hypophosphatemia as a common complication [9]. Our data demonstrating that patients with lower serum phosphate received greater replacement suggests clinician-driven responsive management, though the observation that many patients received replacement despite normal or elevated phosphate levels indicates potential for protocol optimization. Excess phosphate replacement with normal phosphate levels was also observed, suggesting overtreatment, prophylactic treatment or discordance between testing serum phosphate levels and administration of phosphate therapies. Further refinement of replacement strategies could be investigated given the variety of measured serum phosphate levels that were replaced.

### 4.3. Clinical Implications

Several clinical implications emerge from these findings. Firstly, the strong association between standard base excess and hypophosphatemia development suggests that patients presenting with more severe metabolic acidosis warrant heightened vigilance for phosphate depletion, potentially justifying prophylactic or early empiric replacement strategies. This association may allow clinicians to risk-stratify patients at ICU admission and implement targeted monitoring protocols.

The characteristic day 2 nadir observed in this cohort supports a recommendation for serial phosphate monitoring extending beyond the initial presentation, as the most significant phosphate depletion occurs after insulin therapy has been established. This temporal relationship reinforces the importance of viewing hypophosphatemia as a treatment-related complication rather than solely a presenting feature of DKA, with implications for the timing of replacement initiation.

While causality cannot be established from these observational data, phosphate replacement is inexpensive in the context of DKA management, readily available, and carries minimal risk when administered appropriately, suggesting a low threshold (e.g., replacement at mild rather moderate hypophosphatemia) for both supplementations and that potentially pre-emptive replacement in patients with more severe DKA could be investigated.

Importantly, although mortality was not significantly different across phosphate severity groups, the finding of a single patient with extreme hypophosphatemia (<0.1 mmol/L) who experienced ventricular tachycardia serves as a reminder that severe hypophosphatemia carries potentially fatal complications, including respiratory failure, cardiac dysfunction, and neurological impairment [24,25,26]. The rarity of such events should not diminish attention to an easily correctable electrolyte abnormality.

Given the near-universal requirement for insulin therapy in DKA and the mechanistic link between insulin administration and phosphate depletion, early or prophylactic phosphate supplementation may warrant prospective evaluation in future clinical trials.

## 5. Limitations

Several limitations of this study warrant consideration. First, the retrospective design utilizing pre-existing administrative and clinical databases introduces potential for missing data, coding inaccuracies, and unmeasured confounders. The reliance on ICD-10-AM coding for case identification may have excluded patients with DKA not captured by these specific codes or included misclassified cases.

Data regarding insulin administration and other treatments delivered prior to ICU admission (including in the emergency department) were not available. Given that emergency department length of stay was approximately 4 h in our cohort and insulin therapy is typically initiated early in DKA management, a substantial proportion of insulin exposure may have occurred before ICU monitoring commenced and may have affected the observed hypophosphatemia [27,28,29]. This limitation may have contaminated the observed association between insulin dosing and hypophosphatemia [30].

Phosphate monitoring frequency was not standardized across sites or patients, potentially introducing surveillance bias wherein patients with more severe illness received more frequent testing and thus had greater opportunity for hypophosphatemia detection [31]. Similarly, replacement protocols were not standardized, reflecting real-world practice variation but limiting conclusions about optimal replacement strategies [32,33].

While this study encompassed a geographically and clinically diverse cohort of Queensland ICUs, generalizability to other healthcare systems with different patient populations, DKA management protocols, or phosphate replacement practices may be limited. The predominance of type 1 diabetes mellitus in this cohort (75.1%) may also limit applicability to populations with higher proportions of type 2 diabetes- related DKA. There exists possible selection bias for those with ICU admission criteria and possible confounding by illness severity. Compared to DKA admissions requiring ICU admission, non-ICU DKA presentations are generally more well with less severe ketoacidosis; thus, the hypophosphatemia outcomes may not be applicable to mild– moderate cases of DKA.

Finally, the retrospective observational descriptive design does not provide any scope for causal inference. The relationship between hypophosphatemia and ICU length of stay may reflect confounding by DKA severity or other factors, rather than a direct effect of phosphate depletion on recovery trajectory.

## 6. Conclusions

Hypophosphatemia is a common and predictable complication of DKA in patients requiring intensive care admission, occurring in nearly half of all admissions in this large multicenter cohort. Hypophosphatemia characteristically reaches nadir on day 2 of ICU admission and resolves by day 4 with appropriate replacement, though more severe derangements have potential for prolonged ICU stays. These findings suggest early phosphate monitoring and potentially a low threshold for phosphate replacement pending randomized trial evidence.

## Figures and Tables

**Table 1:** Hypophosphatemia demographics a cross all severity subgroups.

	None (>0.80 mmol/L) (n = 436)	Mild (0.50–0.79 mmol/L) (n = 220)	Moderate (0.30–0.49 mmol/L) (n = 124)	Severe (<0.30 mmol/L) (n = 62)	*p*-Value (All Groups, Pairwise)
Demographics					
Age (years) ^1,2^	44 (27, 55)	45 (27, 58)	42 (32, 57)	38 (27, 49)	0.33
BMI (kg/m^2^) ^1,2^	23.3 (20.1, 27.6)	24.1 (20.8, 27.8)	26.5 (21.6, 27.8)	25.3 (20.2, 27.6)	0.01
Hospital Length of Stay Before ICU (h) ^1,2^	3.2 (1.3, 5.5)	3.5 (1.4, 5.5)	3.2 (1.2, 5.3)	5.0 (2.8, 6.8)	0.01
APACHE II ^1,2^	17 (14, 23)	18 (14, 24)	18 (15, 23)	19 (14, 28)	0.13
APACHE III ^1,2^	51 (39, 68)	52 (39, 76)	51 (41, 70)	61 (38, 86)	0.26
Female ^3^	224 (54%)	122 (55%)	68 (55%)	39 (63%)	0.34
Comorbidities					
Respiratory Disease ^4^	7 (1.6%)	2 (0.9%)	3 (2.4%)	0 (0%)	0.61
Ischemic Heart Disease ^4^	28 (6.4%)	14 (6.4%)	4 (3.2%)	4 (6.5%)	0.60
Heart Failure ^4^	13 (3.0%)	5 (2.3%)	5 (4.0%)	2 (3.2%)	0.78
Peripheral Vascular Disease ^4^	4 (0.9%)	3 (1.4%)	0 (0%)	2 (3.2%)	0.22
Cerebrovascular Disease ^4^	0 (0%)	3 (1.4%)	5 (4.0%)	2 (3.2%)	<0.001
Hematological Malignancy ^4^	1 (0.2%)	1 (0.5%)	0 (0%)	0 (0%)	1.00
Cirrhosis (Moderate Severe Liver Disease) ^4^	1 (0.2%)	1 (0.5%)	0 (0%)	2 (3.2%)	0.05
Metastatic Cancer ^3^	4 (0.9%)	1 (0.5%)	2 (1.6%)	1 (1.6%)	0.44
Immunosupression ^3^	9 (2.1%)	10 (4.5%)	3 (2.4%)	0 (0%)	0.18
T1 DM ^3^	329 (75%)	164 (75%)	94 (76%)	45 (73%)	0.96
T2 DM ^3^	89 (20%)	50 (23%)	28 (23%)	14 (23%)	0.89
Not Specified T1DM/T2DM ^4^	18 (4.1%)	6 (2.7%)	2 (1.6%)	3 (4.8%)	0.46
Admission Details					
Emergency Department Admission ^4^	435 (100%)	220 (100%)	122 (98%)	60 (97%)	0.15
Post Elective Surgery ^4^	0 (0%)	0 (0%)	0 (0%)	0 (0%)	1.00
Post Rapid Response Event ^4^	9 (2.1%)	3 (1.4%)	7 (5.7%)	1 (1.7%)	0.10
Goals of Care					
Full Active Treatment ^4^	421 (97%)	215 (98%)	123 (99%)	62 (100%)	0.28
Treatment Limitation Order ^4^	15 (3.4%)	5 (2.3%)	1 (0.8%)	0 (0%)	0.28
Presenting Arterial Blood Gas Values					
pH ^1,2^	7.36 (7.30, 7.40)	7.34 (7.26, 7.4)	7.26 (7.18, 7.38)	7.17 (7.05, 7.25)	<0.001
PaO_2_ (mmHg) ^1,2^	101 (79, 133)	97 (77, 135)	103 (79, 132)	113 (85, 150)	0.30
PCO_2_ (mmHg) ^1,2^	40 (34, 45)	36 (30, 43)	26 (18, 34)	18 (13, 27)	<0.001
Bicarbonate (mmol/L) ^1,2^	23 (19, 25)	20 (15, 23)	12 (7, 19)	6 (4, 11)	<0.001
Base Excess (mmol/L) ^1,2^	−2 (−6, 1)	−5 (−11, −1)	−15 (−20, −7)	−20 (−25, −13)	<0.001
Anion Gap (mmol/L) ^1,2^	8 (5, 10)	9 (7, 13)	14 (8, 21)	18 (13, 22)	<0.001
Glucose (mmol/L) ^1,2^	7 (6, 9)	8 (6, 11)	13 (9, 21)	16 (11, 26)	<0.001

^1^ Median (IQR), ^2^ Kruskal– Wallis, ^3^ Chi-Squared t est, ^4^ Fischer t est, Body Mass Index—BMI, Acute Physiology and Chronic Health Evaluation—APACHE, DM—diabetes mellitus.

**Table 2:** Total and daily phosphate replacement a cross ICU admission.

	None (>0.80 mmol/L) (n = 436)	Proportion Receiving Phosphate Replacement (%, n/N)	Mild (0.50–0.79 mmol/L) (n = 220)	Proportion Receiving Phosphate Replacement (%, n/N)	Moderate (0.30–0.49 mmol/L) (n = 124)	Proportion Receiving Phosphate Replacement (%, n/N)	Severe (<0.30 mmol/L) (n = 62)	Proportion Receiving Phosphate Replacement (%, n/N)	*p*-Value (All Groups, Pairwise)
IV Dose Total (mmol) ^1,2^	0.4 (3.1)	2.3%, 10/436	52.0 (485.2)	37.7%, 83/220	117.9 (738.1)	83.9%, 104/124	64.2 (45.8)	91.9%, 57/62	<0.001
PO Dose Total (mmol) ^1,2^	1.0 (8.4)	1.8%, 8/436	33.0 (64.7)	38.6%, 85/220	50.6 (87.0)	43.5%, 54/124	66.3 (98.0)	54.8%, 34/62	0.05
Day 1 Total (mmol) ^1,2^	0.6 (5.3)	2.3%, 10/436	3.3 (11.5)	11.8%, 26/220	9.1 (20.4)	27.4%, 34/124	16.3 (42.3)	33.9%, 21/62	<0.001
Day 2 Total (mmol) ^1,2^	0.6 (4.0)	2.2%, 9/401	17.1 (71.3)	36.6%, 78/213	33.3 (42.0)	71.8%, 89/124	59.5 (59.0)	86.9%, 53/61	<0.001
Day 3 Total (mmol) ^1,2^	0.2 (2.3)	1.0%, 2/197	20.1 (79.5)	41.3%, 69/167	46.3 (207.5)	64.2%, 61/95	39.4 (35.9)	85.7%, 36/42	<0.001
Day 4 Total (mmol) ^1,2^	0.7 (4.3)	2.9%, 3/104	9.4 (16.8)	33.9%, 39/115	30.4 (127.4)	48.4%, 30/62	25.4 (32.2)	53.6%, 15/28	<0.001
Day 5 Total (mmol) ^1,2^	0.0 (0.0)	0.0%, 0/52	7.6 (17.0)	24.4%, 21/86	48.2 (161.7)	44.7%, 21/47	20.3 (25.0)	59.1%, 13/22	<0.001

^1^ Mean (Standard Deviation), ^2^ Welch T Test. IV—intravenous, PO—Per Os, ICU—Intensive Care Unit.

**Table 3:** Outcomes of hypophosphatemia for e ach severity subgroup.

Variable	None (>0.80 mmol/L) (n = 436)	Mild (0.50–0.79 mmol/L) (n = 220)	Moderate (0.30–0.49 mmol/L) (n = 124)	Severe (<0.30 mmol/L) (n = 62)	*p*-Value (All Groups, Pairwise)
Lowest Phosphate During Admission (mmol/L) ^1,2^	1.09 (0.94, 1.3)	0.66 (0.59, 0.73)	0.41 (0.36, 0.46)	0.22 (0.19, 0.28)	<0.001
Day of Lowest Phosphate (Day) ^1,2^	2 (1, 3)	3 (2, 3)	2 (2, 3)	2 (2, 2)	<0.001
Number of Days with Low Phosphate (Days) ^1,2^	0 (0, 0)	1 (1, 2)	2 (1, 3)	2 (1, 3)	<0.001
Proportion of Days with Low Phosphate (%) ^1,2^	0.00 (0.00, 0.00)	0.33 (0.20, 0.50)	0.50 (0.33, 0.67)	0.50 (0.50, 0.67)	<0.001
ICU LOS (hours) ^1,2^	67 (51, 89)	68 (49, 89)	70 (52, 97)	70 (54, 120)	0.07
Hospital LOS (days) ^1,2^	5.0 (4.0, 8.0)	5.0 (4.0, 9.0)	6.0 (4.0, 9.0)	6.0 (4.0, 11.0)	0.13
ICU Case Fatality (%) ^3,4^	2 (0.6%, n = 349)	1 (0.5%, n = 196)	0 (0.0%, n = 114)	0 (0.0%, n = 57)	1.00
Hospital Case Fatality (%) ^3,4^	4 (1.1%, n = 349)	1 (0.5%, n = 196)	0 (0.0%, n = 114)	0 (0.0%, n = 57)	0.73
30-Day Fatality (%) ^3,4^	7 (2.0%, n = 349)	3 (1.5%, n = 196)	2 (1.8%, n = 114)	0.0 (0.0%, n = 57)	0.94
90-Day Fatality (%) ^3,4^	16 (4.6%, n = 349)	9 (4.6%, n = 196)	5 (4.4%, n = 114)	0 (0.0%, n = 57)	0.44

^1^ Median (IQR), ^2^ Kruskal–Wallis, ^3^ Fischer exact, ^4^ adjusted for readmissions and unique patients. ICU—Intensive Care Unit, LOS—length of stay.

**Table 4:** Hypophosphatemia linear regression table with calculated R^2^ value.

Variable	Univariate Beta (β)	Univariate 95% CI	Univariate *p*-Value	Multivariate Beta (β)	Multivariate 95% CI	Multivariate *p*-Value	Multivariate Partial R^2^ Value
Standard Base Excess (SBE)	0.02	0.01, 0.03	<0.001	0.02	0.01, 0.03	<0.001	0.198
Insulin Dose within 24 h (U/kg/h, Scaled 10×)	−0.14	−0.24, −0.03	0.01	0.02	−0.08, 0.12	0.72	<0.001
Age	0.00	−0.01, 0.00	0.53	0.00	0.00, 0.00	0.45	0.003
Sex (Female)	−0.13	−0.27, 0.01	0.07	−0.07	−0.20, 0.07	0.31	0.010
Weight (kg)	0.00	0.00, 0.01	0.81	0.00	0.00, 0.00	0.48	0.004
Type 1 Diabetes	0.14	−0.29, 0.57	0.51	0.21	−0.18, 0.60	0.29	0.024
Type 2 Diabetes	−0.04	−0.50, 0.42	0.87	0.05	−0.37, 0.46	0.82	0.024
Charlson Comorbidity Index (versus 0–2)	−0.05	−0.20, 0.10	0.50	−0.11	−0.25, 0.04	0.15	0.016

## Data Availability

The datasets presented in this article are not readily available due to privacy and confidentiality regulations. Data released for the purposes of research under section 280 of the Public Health Act 2005 requires an application to the Director-General of Queensland Health (PHA@health.qld.gov.au).

## Supplementary Materials

The following supporting information can be downloaded at: https://www.mdpi.com/article/10.3390/medsci14020249/s1, Figure S1: Diagram of unique patients and unique admissions (Strengthening the reporting of observational studies in epidemiology [STROBE] Checklist). Figure S2: The measured lowest daily phosphate level (mmol/L) versus the total daily phosphate replacement (mmol) (2a–e) or insulin dose (Units/kg/day) (2f–j) on Day 1 through 5 of ICU admission. LOESS trendline is shown in blue. A vertical red line indicates a serum phosphate level of 0.81 mmol/L for the normal reference range. Table S1: Strengthening the reporting of observational studies in epidemiology (STROBE) Checklist.

## Abbreviations

The following abbreviations are used in this manuscript:

AUC	Area Under Curve
APACHE	Acute Physiology and Chronic Health Evaluation
BMI	Body Mass Index
CCI	Charlson Comorbidity Index
DKA	Diabetic Ketoacidosis
DM	Diabetes Mellitus
ICU	Intensive Care Unit
IQR	Inter Quartile Range
IV	Intravenous
LOESS	Locally Estimated Scatterplot Smoothing
LOS	Length of Stay
PO	Per Oral
